# The Recent Developments in Biobased Polymers toward General and Engineering Applications: Polymers that Are Upgraded from Biodegradable Polymers, Analogous to Petroleum-Derived Polymers, and Newly Developed

**DOI:** 10.3390/polym9100523

**Published:** 2017-10-18

**Authors:** Hajime Nakajima, Peter Dijkstra, Katja Loos

**Affiliations:** Macromolecular Chemistry and New Polymeric Materials, Zernike Institute for Advanced Materials, University of Groningen, Nijenborgh 4, 9747 AG Groningen, The Netherlands; hnkajima@gmail.com (H.N.); peter.dijkstra@rug.nl (P.D.)

**Keywords:** biobased polymers, biodegradable polymers, polylactides (PLA), poly(hydroxy alkanoates) (PHAs), bio-poly(ethylene terephthalate) (bio-PET), poly(ethylene 2,5-furandicarboxylate) (PEF), biobased polyamides, succinate polymers, polyterpenes, modified lactide

## Abstract

The main motivation for development of biobased polymers was their biodegradability, which is becoming important due to strong public concern about waste. Reflecting recent changes in the polymer industry, the sustainability of biobased polymers allows them to be used for general and engineering applications. This expansion is driven by the remarkable progress in the processes for refining biomass feedstocks to produce biobased building blocks that allow biobased polymers to have more versatile and adaptable polymer chemical structures and to achieve target properties and functionalities. In this review, biobased polymers are categorized as those that are: (1) upgrades from biodegradable polylactides (PLA), polyhydroxyalkanoates (PHAs), and others; (2) analogous to petroleum-derived polymers such as bio-poly(ethylene terephthalate) (bio-PET); and (3) new biobased polymers such as poly(ethylene 2,5-furandicarboxylate) (PEF). The recent developments and progresses concerning biobased polymers are described, and important technical aspects of those polymers are introduced. Additionally, the recent scientific achievements regarding high-spec engineering-grade biobased polymers are presented.

## 1. Introduction

In the mid-20th century, the polymer industry completely relied on petroleum-derived chemistry, refinery, and engineering processes. The negative impacts of these processes on the environment was scientifically discussed in this period, but the processes were not changed in industrial settings until their negative effects reached a critical level around the 1980s. At this point, biodegradable polymers such as polylactides (PLA), poly(hydroxy alkanoates) (PHAs) succinate derived polymers, and others began to develop, and practical biodegradable polymers were commercialized and launched, solving many waste problems in the agricultural, marine fishery, and construction industries, among others [1,2]. The development of biodegradable polymers is recognized as one of the most successful innovations in the polymer industry to address environmental issues.

Since the late 1990s, the polymer industry has faced two serious problems: global warming and depletion of fossil resources. One solution in combating these problems is to use sustainable resources instead of fossil-based resources. Biomass feedstocks are a promising resource because of their sustainability. Although biomass is the oldest energy source, having been used for direct combustion since the Stone Age, it is still uncommon to utilize biomass as chemical building blocks and fuel during refinery processes [3]. The development of refinery processes has been dramatically accelerated due to improvements in the combinations of chemical and biological pathways for production of, for example, bio-ethanol, bio-diesel, and bio-olefins [4,5,6]. Biomass feedstock can be converted into raw materials for polymer production, and the resulting polymers are called “biobased polymers” [7,8,9,10]. As the term “biobased polymers” is still relatively new in polymer science and industry, it is sometimes confused with other terms such as biopolymers, biodegradable polymers, and bioabsorbable polymers. More specifically a biopolymer is classified as a natural polymer formed by plants, microorganisms, and animals. Naturally derived biomass polymers are termed “1st class biobased polymers” for and bio-engineered polymers (vide infra) as “2nd class biobased polymers”. Biopolymers show biodegradability, but this class of polymers does not include artificially synthesized biodegradable polymers. Biodegradable polymers include both naturally derived ones and artificially synthesized ones. They are sometimes defined as biocompostable polymers, especially in waste, agricultural, fishery and construction industries. The term biodegradable polymer is also used for medical, pharmaceutical, and bioengineering applications. Biodegradable polymers consisting of naturally derived building blocks are also called bioabsorbable polymers, when they are specifically applied for medical, pharmaceutical, or other bioengineering applications.

The importance of biobased polymers is well known, and much research and development activities concerns the use of biobased polymers in science, engineering, and industry. Generally, biobased polymers are classified into three classes:

- 1st class; naturally derived biomass polymers: direct use of biomass as polymeric material including chemically modified ones such as cellulose, cellulose acetate, starches, chitin, modified starch, etc.;
- 2nd class; bio-engineered polymers: bio-synthesized by using microorganisms and plants such as poly(hydroxy alkanoates (PHAs), poly(glutamic acid), etc.;
- 3rd class; synthetic polymers such as polylactide (PLA), poly(butylene succinate) (PBS), bio-polyolefins, bio-poly(ethylene terephtalic acid) (bio-PET) [8,9].

Usually, 1st class is directly used without any purification and 2nd class polymers are directly produced from naturally derived polymers without any breakdown, and they play an important role in situations that require biodegradability. Direct usage of 1st and 2nd class polymers allows for more efficient production, which can produce desired functionalities and physical properties, but chemical structure designs have limited flexibility. Monomers used in 3rd class polymers are produced from naturally derived molecules or by the breakdown of naturally derived macromolecules through the combination of chemical and biochemical processes. As breakdown processes allow monomers to have versatile chemical structures, polymers comprised of these monomers also have extremely versatile chemical structures. It is practically possible to introduce monomers in 3rd class polymers into the existing production system of petroleum-derived polymers. For the above reasons, the 3rd class of biobased polymers is the most promising. Some of these 3rd class polymers such as bio-polyolefines and bio-PET are not supposed to enter natural biological cycles after use. Thus, the contribution for reducing environmental impact from these polymer classes is mainly derived from reducing the carbon footprint. In Table 1, the chronological development and categorization of biobased polymers that are based on application fields are displayed and compared with those of petroleum-derived polymers. From 1970 to 1990, PLA (low l-content) and poly(hydroxy alkanoates) (PHAs) are the most important and representative development of biobased polymers [11]. During that period, scientists developed a fundamental understanding of biobased polymers for future applications. Since the 1990s, biobased polymers have gradually shifted from biodegradable applications to general and engineering applications. High l-content PLLA, high molecular weight PHAs, and stereocomplexed-PLA (sc-PLA) (low *T*_m_ grade) are important examples of this development. The deliverables of these were effectively applied to industrialize general applications of PLLA, PHAs, and succinate polymers. In this period, high-performance new-generation PLAs, such as sc-PLA (high *T*_m_ grade) [12] and stereoblock-PLA (sb-PLA) [13], were proactively created. After the successful upgrading of these biodegradable polymers, more promising building blocks have been identified to create more attractive chemical structures for biobased polymers [6], which are known as the US Department of Energy’s (DOE’s) 12 top biobased molecules [14]. Around 2010, engineering-grade biobased polymers that were analogous to petroleum-derived polymers such as poly(ethylene terephthalate) (PET) and polyamides began to be applied to industry. Completely new biobased polymers with the potential for super-engineering applications also started to appear around 2010. It is expected these will be applied to new applications and conventional petroleum-derived polymers will rarely be used in the distant future.

Recent economic studies have revealed that biobased polymers can create new business opportunities and stable growth in new, plastic markets [15,16]. Actual growth is influenced by the current events and issues concerning economics, politics, and international affairs, but stable growth of the biobased polymer industry was observed in all proposed scenarios [15]. The strong social interest in a sustainable society is still the most important factor in the development of these polymers, but recent improvements in the quality and functionality of biobased polymers have led the growth of these plastic markets. There are several successful examples of industrialization of these polymers, including pilot-scale production of polylactide (PLA) at NatureWorks and Corbion/Total; poly(trimethylene terephthalate) (PTT) at DuPont; poly(isosorbide carbonate) at Mitsubishi Chemicals; biobased polyamides at Arkema, Toray, BASF, DSM, and others; and poly(ethylene 2,5-furandicarboxylate) (PEF) at Synvina.

Biobased polymers are being applied to general and engineering situations. For example, because of improvements in the physical durability and processability of PLA, it has been used in the packaging industry [17,18,19]. In addition, due to the superior gas barrier properties of PEF, it is being used for bottles, films, and other packaging materials in the food and beverage industry [20,21]. Further, biobased PTT is analogous to petroleum-derived PTT, and its biobased, sustainable nature and intrinsic flexible chain properties allow comfortable stretching and shape recovery properties are attractive promising [22]. However, the stability of the production and processability of these biobased polymers can still be improved. The current general approach to improving processability is physical modification and optimization of polymer processing, including optimization of processing parameters, extruder screw design, selection of appropriate additives, and post-orientation for strain-induced crystallization. These developments in processing conditions have made biobased polymers analogous to certain petroleum-derived polymers. In addition to physical modification and optimization, the importance of chemical modification and optimization has been emphasized as they allow for further improvement and new functionalities of biobased polymers. This review introduces recent important developments in chemical modifications of biobased polymers and development of new biobased building blocks for new generation biobased polymers.

## 2. Biobased Polymers: Upgraded from Biodegradable-Grade Polymers

PLA, PHAs, and succinate polymers are the most common biobased polymers since they have been successfully applied in the biodegradable plastic industry. The biodegradability of these polymers has been utilized to solve environmental issues, such as waste and public pollution. Due to changes in the social requirements for biodegradable polymers, it is necessary to improve the performance of biodegradable polymers so they can be used for general and engineering applications. The recent examples of development and applications of PLA, PHAs, and succinate polymers are described. Their fundamental properties and chemistries are also introduced.

### 2.1. Polylactide (PLA)

#### 2.1.1. High l-Content PLA (PLLA)

**Figure 1:**
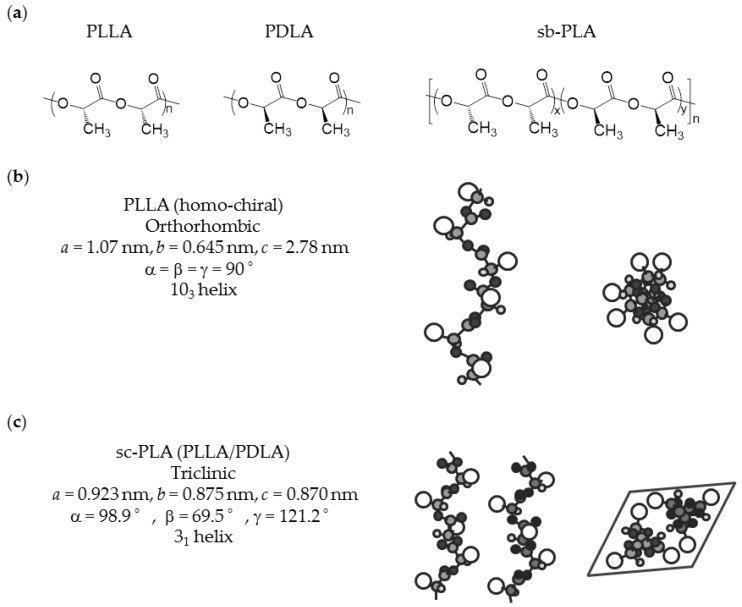
Chemical structures and conformation of PLA: (**a**) chemical structures and chirality; (**b**) conformation of PLLA (homo-chiral) [33]; and (**c**) conformation of sc-PLA from a combination of PLLA and PDLA [34].

PLA is generally prepared via ring-opening polymerization (ROP) of lactide, which is a cyclic dimer from lactic acid. Direct polycondensation from lactic acid is also performed, but ROP is the standard process in most industries. PLA has a chiral active chain structure, and controlling it allows one to determine the physical properties of PLA. The relationship between the physical properties and l-unit content of PLA has been comprehensively studied [23]. Regarding the effectiveness of biological production, l-lactic acid has superior productivity compared to d-lactic acid. Therefore, poly(l-lactide) (PLLA) is more commonly commercialized. The parameters listed in Table 2 are related to crystallinity. The table shows a clear trend in which the physical properties of PLA are improved by increasing the purity of the l-unit content. The growth rate of spherulite and increase in l-unit content are almost proportional; when the l-unit content is increased 1.0%, the growth rate of spherulite is increased about 2.0 times. Other parameters concerning the crystallization properties of PLA show a similar trend; crystallinity depends on crystallization conditions, but in this report, it is described that crystallinity increases 1.3 times when l-unit content is increased 1.0%. The common crystal structure of highly pure homo-chiral PLA is pseudo-orthorhombic and consists of left-handed 10_3_-helical chains, which are generally called α-forms [24,25,26]. A slightly disordered pseudo-orthorhombic PLA is called an α’-form [27,28]. Because of the slightly disordered structure of the α’-form, an α’-form based PLA crystal has lower thermal and physical properties than those of α-form. Table 3 summarizes the infrared spectroscopy (IR) frequencies of α-forms and α’-forms. Although both α-forms and α’-forms have the same helical conformation, IR analysis of these forms reveals different results, which can be utilized for detection of crystallization form of PLA [29]. The chemical structure and conformation of homo-chiral PLLA are shown in Figure 1.

#### 2.1.2. Stereocomplexed PLA

Sc-PLA is a complex form of PLLA and poly(d-lactide) (PDLA) that was initially reported as an insoluble precipitant for solutions [30]. As the chemical properties of PLA change during the formation of sc-PLA, the original solubilities of homo-chiral PLAs are lost. As a result, sc-PLA is selectively precipitated as granules made from sc-PLA crystallites. A sc-PLA film is created from the Langmuir–Blodgett membrane when PLLA and PDLA are combined [31]. In addition, PLLA and PDLA with molecular weights as high as 1000 kDa have preferable stereocomplexation on the water surface. Further, sc-PLA is assembled on a quartz crystal microbalance (QCM) substrate by stepwise immersion of the QCM in acetonitrile solutions of PLLA and PDLA [32]. The Langmuir–Blodgett membrane and assembled methods are interesting new approaches to achieve nano-ordered structural control of sc-PLA layers.

A striking property of sc-PLA is its high *T*_m_ (around 230 °C). This is 50 °C higher than the conventional *T*_m_ of homo-chiral high l-content PLLA. In contrast to the stereocomplexation of high molecular weight PLA in a solution state, a simple polymer melt-blend of PLLA and PDLA is usually accompanied by homo-chiral crystallization of PLLA and PDLA, particularly when their molecular weight is sufficient for general industrial applications.

The homo-chiral crystals deteriorate the intrinsic properties of sc-PLA, but this drawback can be overcome using sb-PLA. A sb-PLA with an equimolar or moderate non-equimolar PLLA to PDLA ratio features 100% selective stereocomplexation [13]. Therefore, formation of homo-chiral PLA-derived crystallization, which is known to cause poor physical performance of sc-PLA produced from direct combination of PLLA and PDLA, is prevented. An important issue with sb-PLA is that its *T*_g_ is identical to that of homo-chiral PLA, and thus the final thermal durability of sb-PLA is controlled by *T*_g_ due to its relatively low crystallinity. The chemical structure of sb-PLA and conformation of sc-PLA from a combination of PLLA/PDLA are shown in Figure 1.

#### 2.1.3. Examples of PLA Applications

Although the application of PLA was limited to biodegradable plastics in the early stage of its development, it has been successfully applied to general and semi-engineering situations and achieved successful commercialization. The most common commercialized PLA in the world is made by NatureWorks, which trademarked their PLA “Ingeo” [35]. Currently, there are more than 20 commercialized types of Ingeo with both amorphous and semi-crystalline structures, allowing customers to choose the PLA that is appropriate for their specific situations (Table 4). Another important player affecting the industrialization of PLA is Corbion/Total. Now, there are many commercial-grade PLAs on the market, such as Biofoam, made by Synbra; Revode, made by Zhejiang Hisun Biomaterials Biological Engineering; Futerro, made by Futerro; Lacea, made by Mitsu Chemicals; and Terramac, made by Unitika. sc-PLA will play a key role in future engineering applications of PLA. Biofront, made by Teijin, is a good example of the industrial development of sc-PLA [36]. This product features high physical properties, including a melting point of 215 °C, HDT of 130 °C to 0.45 MPa, and a modulus of 115 MPa at 23 °C. These properties are considered suitable enough for sc-PLA to replace petroleum-derived engineering plastics.

### 2.2. Poly(hydroxyalkanoates) (PHAs)

PHAs are members of a family of polyesters that consist of hydroxyalkanoate monomers. In nature, they exist as homopolymers such as poly(3-hydroxybutyrate) (P3HB) or copolymer poly(3-hydroxybutyrate-*co*-3-hydroxyvalerate) (P(3HB-*co*-3HV)) [37]. PHAs exist as granules of pure polymer in bacteria, which are used as an energy storage medium (akin to fat for animals and starch for plants). PHAs are commercially produced using energy-rich feedstock, which is transformed into fatty acids on which the bacteria feed. During industrial production of PHAs, after a few “feast–famine” cycles, cells are isolated and lysed. The polymer is extracted from the remains of the cells, purified, and processed into pellets or powder [37]. In addition to using pure feedstock as a source of energy for PHAs production, there are on-going efforts to use energy-rich waste water as feedstock and thus as PHAs [38]. Production of PHAs can be improved using genetic modification, either by increasing the amount of PHAs-producing bacteria or by modifying plants to start making PHAs [39,40]. As chemical synthesis of PHAs via the ROP of a corresponding lactone is feasible, ROP of lactones for PHAs can be done via metal-based or enzymatic catalysts [41]. However, the chain of chemically synthesized PHAs is shorter in length than that of biologically synthesized PHAs. The latter also ensures great stereo control and enantiomeric pure (*R*) configuration in almost all PHAs. Through depolymerization, enantiomeric purity allows for the creation of an enantiomeric monomer that can be used as a building block [42]. On the other hand, when pure (*S*)-methyl 3-hydroxybutyrate is used as feedstock for the production of PHAs, the corresponding (*S*)-configuration polymer is produced [43].

**Figure 2:**
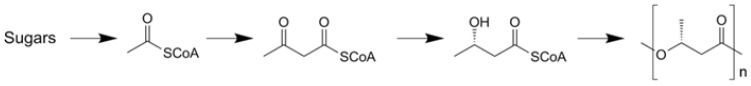
Biological synthesis scheme of P3HB.

The biological synthesis of P3HB is displayed in Figure 2. Sugars in the feedstock are converted to acetates, which are complexed to coenzyme A and form acetyl CoA. This product is dimerized to acetoacetyl A. Additionally, through reduction, hydroxy butyryl CoA is polymerized.

**Figure 3:**
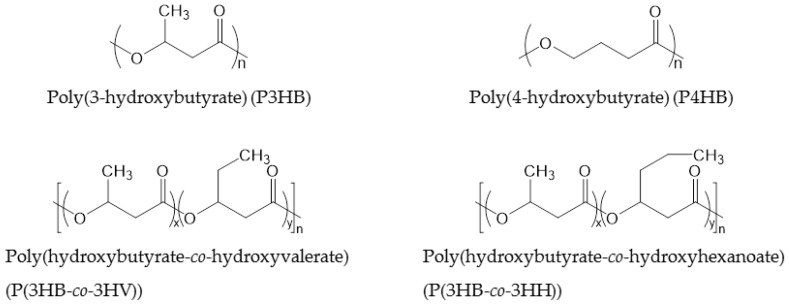
Chemical structures of PHAs.

PHAs consisting of 4–14 carbon atoms in the repeating unit are called “short chain length PHAs” (sCL-PHAs) or “medium chain length PHAs” (mCL-PHAs) [44]. Some of these PHAs are commercialized. The average molecular weight (*M*_w_) of PHAs corresponds to their chain length. Typically, the *M*_w_ of sCL-PHAs is around 500,000, while that of mCL-PHAs is lower than 100,000. In large part, the chain length of PHAs determines the flexibility of the polymer, with short chain butyrate providing the most rigidity and longer side chains disturbing crystal packing, resulting in more flexibility. Long chain length PHAs, which consist of repeating units of more than 14 carbon atoms, and PHAs that consist of either aromatic or unsaturated side-chains are rarely commercialized. The most commonly commercialized PHAs are P3HB, P(3HB-*co*-3HV) and P(3HB-*co*-3-hydroxyhexanoate) (P(3HB-*co*-3HH)), the thermal and physical properties of which are displayed in Table 5. P3HB has a *T*_g_ of 4 °C, which becomes lower when the PHAs has a longer chain length. The *T*_m_ of PHAs decreases with increasing chain length; P3HB has a melt temperature of 160 °C, while the melt temperature of P3HB-*co*-3HV is only 145 °C. Both *T*_g_ and *T*_m_ can be altered by changing the ratio of repeating units. The chemical structures of PHAs are shown in Figure 3.

P3HB crystalizes in an orthorhombic structure (P3HB: a = 5.76 Å, b = 13.20 Å, and c = 5.96 Å), and its crystallinity can reach 80% [46]. Pure P3HB has poor nucleation density, leading to slow crystallization, due to the formation of large crystallites induced by poorly dispersed nucleation points. A promising way to improve crystallization speed is quiescent crystallization in isothermal conditions, which are 10–20 °C lower in temperature than crystallization conditions. This allows crystallization with the most possibility for arranging chains. It should be applied with appropriate nucleation agents for optimum processing in industry. Processing PHAs is challenging compared to conventional petroleum-derived polymers because of their sensitivity to thermal degradation and slow solidification due to slow crystallization. The degradation temperature of PHAs is around 180 °C, which is near the optimum processing temperature for polyester. A rapid increase of shear-induced internal heat can cause severe degradation, leading to a drop in molecular weight and discoloration. For these reasons, it is important to precisely monitor and control the practical temperature in extruders during processing of PHAs. Processing PHAs is challenging also due to their low durability and tackiness in the final product due to insufficient crystallinity. Cooling below the *T*_g_ can easily decrease tackiness, but the *T*_g_ of PHAs is 0 °C or lower, which is not an easily controllable temperature for the conventional extruders and molders used in the plastic industry.

### 2.3. Polysaccharides

**Figure 4:**
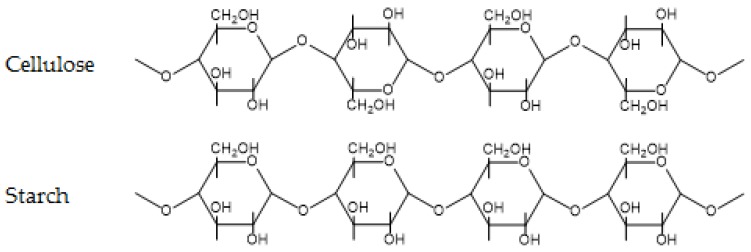
Chemical structure of cellulose and starch.

Carbohydrates are probably the most prevalent group of organic chemicals on earth. Encompassing monosaccharides, disaccharides (commonly known as sugars), oligosaccharides, and polysaccharides, they are present in all lifeforms. Polysaccharides include well known polymers, such as cellulose and starch and their derivatives, as well as more exotic polymers, such as chitosan and pectin. In this review, we will briefly focus on cellulose and starch. The chemical structures of cellulose and starch are shown in Figure 4.

Cellulose, or more specifically, cellulose nitrate, has a special place in the history of polymer chemistry: it is the first polymer to be deliberately synthesized by human beings during the quest for synthetic ivory. Cellulose nitrate resulted in further derivatives of cellulose, such as cellulose acetate because of the safety aspects in handling and processing. Cellulose derivatives are still used on a wide scale in film, cigarette filters, and biomedical applications [47]. Other cellulose products, such as paper and cotton clothes, can be viewed as polymeric products. Cellulose is important to the polymer industry due to its abundance in plant fibers. It is not used as a polymer matrix but as an additive; the incorporation of natural fibers (e.g., wood, hemp, and flax) into a polymer compound improves the mechanical properties of the final product. The current focus of cellulose research is nano-cellulose, including cellulose nano-fibers and nano-crystalline cellulose [48,49,50]. Cellulose nanofibers are delaminated fibrils with a small diameter (5–25 nm) and long length (micrometer scale). Cellulose nano-fibers have interesting properties, such as high tensile strength and absorbance ratio. Nano-crystalline cellulose—tiny crystals of cellulose—is of interest due to its high mechanical load and shear thinning properties. Both materials are produced from wood fibers after intensive physical, chemical, and separation procedures.

As previously mentioned, starch is a means for obtaining and storing energy in plants. Starch-rich plants have been used for ages as sources of food, and starch is very commonly extracted for use in industry. Starch is stored in granules containing linear amylose and branched amylopectin. Both feature repeating units of d-glucose linked in α 1,4 fashion, with amylopectin containing about 6% of 1,6 linkages. A natural starch is not directly applicable for a processing, rather starch and water are passed through an extruder which produces thermoplastic starch (TPS) [51,52]. TPS is however not stable and retrodegradation is an issue; i.e., TPS tries to revert to its natural starch form. The main process hereby is the gelatinization of the starch granules which causes swelling of the amorphous parts of the granules. To stabilize the thermoplastic starch plasticizers (e.g., glycol and sugars) are added. An interesting approach to improve starch processability is a formation of amylose–lysophosphatidylcholine complexation to control rheological behaviors [53]. The induced lower modulus proved the formation of particle gel, resulting in less retrogradation. The complexation is also able to decrease the susceptibility of starch granules against amylase digestion [54,55].

### 2.4. Succinate Polymers

As biobased succinic acid (SA) becomes more commercially available, more biobased succinate polymers are being developed [56,57]. Poly(butylene succinate) (PBS), which is produced by direct polycondensation of SA and butanediol (BD), is one of the most well-known succinate polymers [58]. Both SA and BD for commercialized PBS were only produced from fossil fuel resources until recently, but the high interest in green sources led to the discovery that the two monomers can be obtained from refined biomass feedstock.

**Figure 5:**
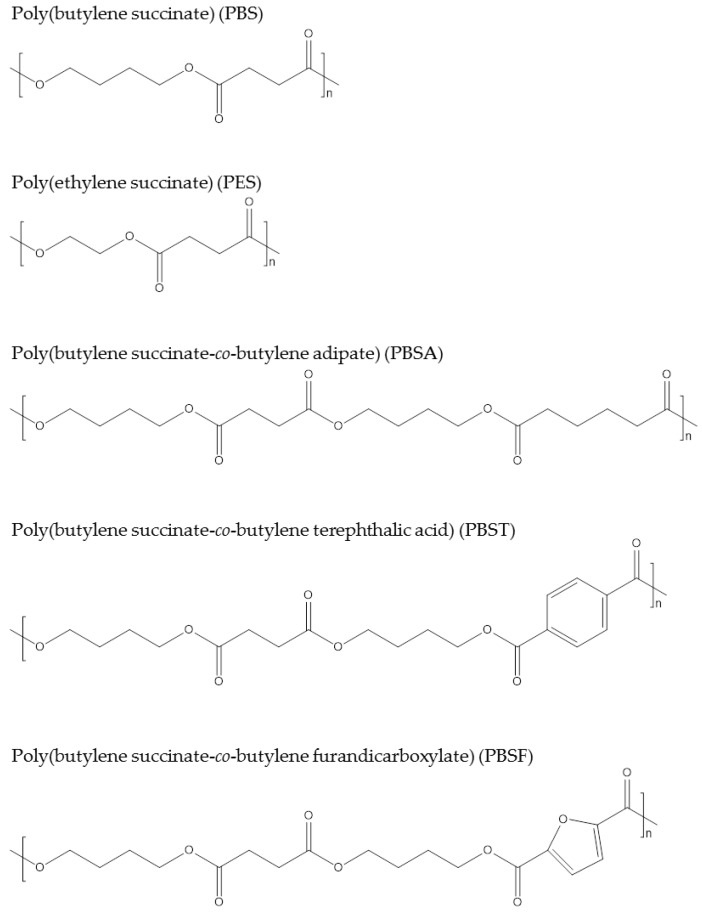
Chemical structures of succinate polymers.

The widely commercialized PBS named “Bionolle” was launched by Showa Denko in 1993, and 3000–10,000 tons are produced per year. Since 2013, Succinity, a joint venture of BASF and Corbion, has been able to produce 10,000 tons of 100% biobased PBS. Poly(ethylene succinate) (PES) produced via polymerization of succinic acid and ethylene glycol is biodegradable and could also be sourced from biobased building blocks [59]. PES was commercialized by Nippon Shokubai from fossil resources. It has been claimed that PES is suited for film applications due to its good oxygen barrier properties and elongation. Copolymers of succinic acid and other dicarboxylic acids, such as adipic acid for poly(butylene succinate-*co*-butylene adipate) [60], poly(butylene succinate-*co*-butylene terephthalic acid) [61], and poly(butylene succinate-*co*-butylene furandicarboxylate) [62], have been reported. Figure 5 shows the chemical structures of the presented succinate polymers. Because of these polymers’ relatively long alkyl chains, they usually have soft properties; for instance, PBS has a *T*_m_ of 115 °C and tensile strength of 30–35 MPa. Thus, succinate polymers are usually considered an alternative to polyolefins in the packaging industry.

## 3. Biobased Polymers Analogous to Conventional Petroleum-Derived Polymers

### 3.1. Biobased Polyethylene (Bio-PE)

Due to soaring oil prices, bio-ethanol produced by fermentation of sugar streams attracted the fuel industry in the 1970s. Bio-ethanol could also be chemically converted to bio-ethylene for production of biobased polyethylene (bio-PE) [63]. A drop in the price of oil diminished the bio-PE market, but the polymer continues to be exploited by important players such as Braskem due to increasing oil prices and environmental awareness [64]. The big advantage of bio-PE is the fact that its properties are identical to fossil-based PE, which has a complete infrastructure for processing and recycling. However, it faces direct competition with fossil-based feedstock, the price of which heavily fluctuates (e.g., shale gas is cheap) [65]. The downside of biobased PE is that it is not biodegradable. However, as will be shown next, some plastics produced from fossil fuel feedstock are biodegradable.

### 3.2. Biobased Poly(Ethylene Terephthalate) (PET) and Poly(Trimethylene Terephthalate) (PTT)

PET is a high-performance engineering plastic with physical properties that are suitable enough to be applied to bottles, fibers, films, and engineering applications. While PET plays an important role in the plastic market, huge consumption of this polymer results in serious environmental issues, especially regarding waste, because of its poor sustainability and degradability. Polymer-to-polymer material recycling of PET has been launched in some fields, but it is always accompanied by non-negligible deterioration of the polymer’s physical properties in the final recycled products due to side-reactions and thermal degradation, hydrolysis, and thermo-oxidative degradation during recycling. Ways to chemically recycle PET are under development, but many technical difficulties, such as the high stability of PET under normal hydrolysis, alcoholysis, or breakdown processes, must be overcome. To realize a truly sustainable PET industry, it is important to establish sustainable production of monomers for biobased PET (bio-PET) from sustainable resources, such as biomass. First, ethylene glycol (EG) from petroleum-derived sources must be replaced by EG from biobased sources. The Coca-Cola Company (TCCC), a beverage giant, has accelerated the production of bio-PET known as “PlantBottle” [66]. PlantBottle, which was launched in 2009, consists of 30% biobased materials, 100% biobased EG (bio-EG) and petroleum-derived terephthalic acid (TPA).

**Figure 6:**
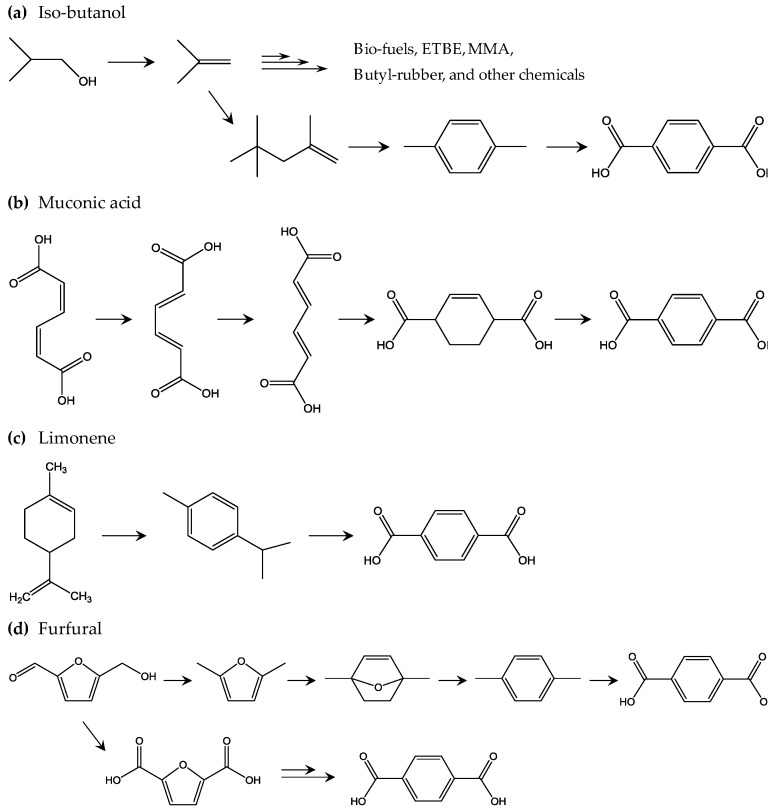
Proposed methods to achieve biobased TPA: (**a**) the *iso*-butanol method [67]; (**b**) the muconic acid method [68]; (**c**) the limonene method [69]; and (**d**) the furfural method [70,71,72,73].

Following this, biobased terephthalic acid (bio-TPA) is being developed to further improve the sustainability of PET, as bio-TPA is produced from naturally derived sustainable biomass feedstock. Theoretically, combining bio-EG and bio-TPA could achieve 100% natural biomass feedstock derived bio-PET. Figure 6 shows the proposed development of bio-TPA from biomass feedstock. One of the most important players in the development of bio-TPA is Gevo [67]. Based on technology announced by Gevo, biobased isobutylene obtained from *iso*-butanol, which is produced by dehydration of sugar, is a key building block in various chemicals, such as ethyl *tert*-butyl ether, methyl methacrylate, isooctane, and other alkanes. For bio-TPA production, *p*-xylene is first produced by cyclization of two isooctane molecules via dehydrogenation. Second, the *p*-xylene is converted to bio-TPA via oxidation. However, this is not the only way to obtain bio-TPA; it has been proposed that bio-TPA could be obtained from other biomass-derived products. Muconic acid, which is produced from sugar through a combination of chemical processes and biorefinery, is one interesting building block for bio-TPA [68]. After a series of stepwise *cis–cis* to *trans–trans* transitions of muconic acid, a tetrahydro terephthalic acid (THTA) can be produced by an ethylene addition reaction, dehydrogenation of which produces bio-TPA. Bio-TPA produced from limonene-derived building blocks is also under development. *p*-cymene is a limonene-derived precursor that can be produced from chemical refinery of limonene [69]. Oxidation uses concentrated nitric acid for the *iso*-propyl group, which reacts with potassium permanganate. This oxidation results in 85% overall conversion from limonene, which is the target in industrial applications. Bio-TPA from furan derivatives should be also featured, as biobased furan derivatives such as 2,5-furan dicarboxylic acid (FDCA) are already in large scale pilot production state [70,71,72,73]. Diels-Alder (DA) reaction is the key chemical reaction in the bio-TPA production from furan derivatives. First, furfural is oxidized and dehydrated to produce maleic anhydride, which is then reacted with furan to produce a DA adduct. Dehydration of the DA adduct results in phthalic anhydride, which is converted to bio-TPA via phthalic acid and dipotassium phthalate. Another interesting bio-TPA synthesis pathway involving DA was reported by Avantium, the leading developer of biobased FDCA. Hydroxymethylfurfural (HMF) from fructose is an important precursor of FDCA and is produced by hydrogenation to convert HMF to dimethyl furan (DMF). DMF is converted to *p*-xylene through several steps, such as cyclization with ethylene by DA and dehydration, and then the *p*-xylene is converted to bio-TPA. Another interesting approach is reported by BioBTX, which is building a pilot plant to produce aromatics (benzene, toluene, and terephthalate or BTX) by means of catalytic pyrolysis of biomass (e.g., wood and other lignin-rich biomass resources) [74]. Figure 6 shows the four methods of bio-TPA production discussed above. Similar to bio-EG, bio-based 1,3-propoane diol (bio-PDO) is used for development of biobased poly(trimethylene terephthalate) (PTT). Because of the good shape recovery properties of PTT due to its unique chain conformation, PTT fibers are used in the carpet and textile industries. Partnering with Tate & Lyle and Genencor, DuPont produces bio-PDO named “Susterra” by fermenting sugars from starches [75]. Susterra is used as building block for biobased PTT named “Sorona”, which consists of 37 wt % sustainable components. Biobased poly(butylene terephthalate) (PBT) will become an available biobased polyester, since biobased production of monomer component BD is under steady development [76]. PBT is used for special applications that require high dimensional stability and excellent slidability. It explores new applications of biobased polymers when PET and PTT are rarely used.

### 3.3. Biobased Polyamides

**Figure 7:**
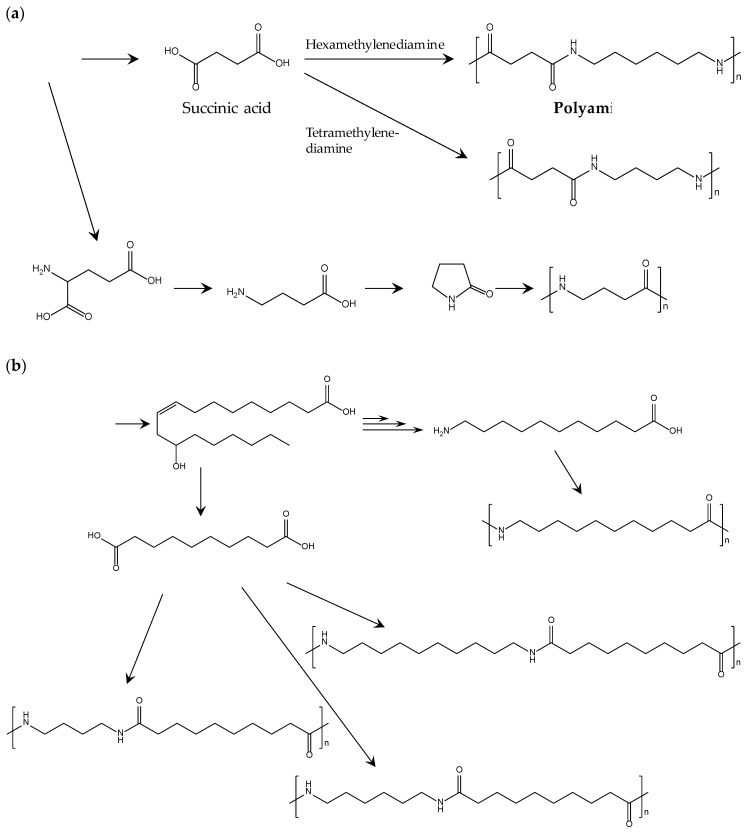
Method of building block production and biobased polyamide polymerization: (**a**) biobased polyamides from sugar; (**b**) from castor oil [78].

Development of biobased polyamides is accelerated by the recent progress in the refinery of biobased building blocks. Table 6 lists the chemical structures, typical *T*_m_, moduli, and suppliers of commercialized biobased polyamides. Polyamides 6 and 6.6 are representative petroleum-derived polyamides that are widely used for many general and engineering applications. Thus, making the properties of biobased polyamides similar to those of polyamides 6 and 6.6 is a reasonable milestone to set when creating realistic development strategies. Figure 7 shows the typical methods producing building blocks and polymerizing biobased polyamides. As shown in Table 6, the thermal properties of biobased polyamides containing four carbons (4C) are comparable or higher than those of polyamides 6 and 6.6. The high *T*_m_ of 4C polyamides is accompanied by high thermal durability and mechanical strength, but the rigidity of these polyamides should be moderated to ensure stable processing in practical extrusion and injection molding.

Among the general techniques for moderation of rigidness, branching in the main chain of a polymer may be the most promising for polyamide 4 [77]. In this report, 3- and 4-arm branched polyamide 4 were prepared with high molecular weight, comparable to linear polyamides. The branched structure improved mechanical properties without decreasing *T*_m_. Although promising improvements were made in physical properties, there are some technical issues regarding polyamide 4 that must be overcome, including the level of gel formation during polymerization. When the amount of initiator for branched polyamide 4 is higher than 3.0 mol %, some gelation occurs, which might negatively affect the physical properties of the final product. For stable industrial production of polyamide 4, the optimum polymerization process must be determined. Table 6 displays polyamides that consist of 4C, 10-carbon (10C), 11-carbon (11C), and 12-carbon (12C) biobased building blocks [77,78,79]. The 10C, 11C, and 12C comprising biobased polyamides have milder and softer physical properties. However, these properties are prized for applications such as automotive fuel lines, bike tubing, and cable coating, which require flexibility. Sebacic acid for C10 and 11-aminoundecanoic acid for C11 are the building blocks for polyamides 4.10, 6.10, 10.10 and 11. The long alkyl chains of these result in low water uptake and low density, which are advantages over conventional polyamides. Besides the relatively low *T*_m_ of polyamides 6.10, 10.10 and 11 compared to polyamides 6 and 6.6, the flexibility of long alkyl chains is attractive for engineering applications that require high impact resistance and resilience.

Another interesting approach to creating biobased polyamides is the development of biobased lactams [80,81,82]. The proposed steps of lactam synthesis are rather complicated, but it is expected that the tunable aspects of biobased lactams will lead to new functionalized polyamides in the near future.

## 4. Newly Developed Biobased Polymers

### 4.1. Poly(Ethylene 2,5-Furandicarboxylate) (PEF)

#### 4.1.1. PEF from Condensation of Diol and FDCA

**Figure 8:**
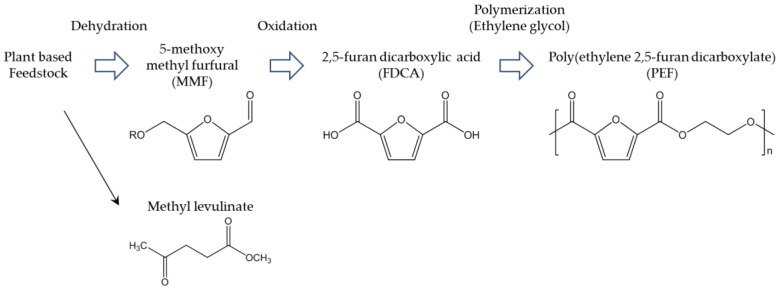
Avantium’s PEF production process [87].

In the past, PEF was not considered special; it was a downgraded PET because of its slow crystallization and low *T*_m_. Although the general flow of polymerization processes, physical properties, and fundamental crystallography of PEF had been reported in the 1940s, the available information was not sufficient for practical application. However, in 2008, some reliable information about PEF, including its currently known polymerizations, was reported [83]. Around the same time, the widely known thermal properties of PEF—*T*_m_ around 210 °C and *T*_g_ around 80 °C—were reported [84]. Other studies followed, increasing the scientific understanding of the physical properties of PEF. The thermal decomposition temperature of PEF is approximately 300 °C, which also results in β-hydrogen bonds [85]. The brittleness and rigidity of PEF result in about 4% elongation at break [86]. PEF is generally produced by polycondensation and polytransesterification of EG and FDCA, derivatives of dichloride-FDCA, dimethyl-FDCA, diethyl-FDCA, or bis-(hydroxyethyl)-FDCA [85]. Solid-state polymerization (SSP) is the key to obtaining high molecular weight, which enables PEF to be suitable for engineering applications. These steps are analogous to industrial processes for producing PET. The results of scientific studies have been successfully applied to pilot and upcoming industrial production of PEF. The most widely known example of industrial PEF production is that of Synvina, a joint venture of Avantium and BASF. Figure 8 shows Avantium’s plan for production of PEF from FDCA derived from fructose [87]. First, fructose is converted to 5-methoxy methyl furfural (MMF) by dehydration. MMF is then treated by oxidation to produce crude FDCA, which can be highly purified to achieve high-grade FDCA that can be used for production of PEF. With optimal modification, MMF and hydromethyl furfural can serve as important intermediate biobased building blocks for fine chemicals. Therefore, the side products of FDCA may create a new biobased industry. In Avantium’s plan, the side product methyl levulinate is also considered an interesting chemical for development of biobased building blocks. The important properties and functionalities of PEF are compared with those of PET in Table 7 [88]. The remarkably high gas barrier properties of PEF should be emphasized; the high O_2_ barrier is advantageous for packaging, leading to PEF’s practical application in the food and beverage industry. Thermal properties of other poly(alkylene furanoates) (PAF) from FDCA and other biobased aliphatic diols such as C3–C18 long alkyl chain liner alkyls are also reported [85,86,89,90,91,92,93,94]. The *T*_m_ and *T*_g_ of them constantly drop, as length of alkylene chain becomes longer, as it is represented by *T*_m_ and *T*_g_ of PEF are 211 °C and 86 °C, poly(trimethylene furanoate) are 172 °C and 57 °C, poly(butylene furanoate) (PBF) are 172 °C and 44 °C, and poly(1,6-hexanediol furanoate) are 144 °C and 13 °C, respectively [92]. PAF consisting of isosorbide which has rigid and bulky structure is also an interesting polymer because of its outstanding *T*_g_ [93]. The reported isosorbide containing PAF shows *T*_g_ 196 °C with excellent amorphous properties. Long alkylene chain containing PAF are also prepared by environmentally benign process i.e., enzymatic polymerization [95]. In this report, the structure–property relationships for example, alkylene chain length and thermal properties, crystallinity, and alkylene component were scientifically discussed and summarized. The enzymatic polymerization was applied for FDCA based polyamide [96] and furan containing polyester from 2,5-bis(hydroxymethyl)furan and aliphatic dicarboxylic acid [97].

#### 4.1.2. PEF from ROP

**Figure 9:**
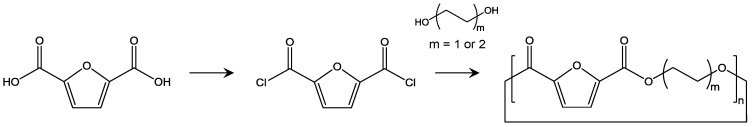
Synthetic scheme of cyclic oligomers for PEF and PBF [98,99].

Although the improvements in polycondensation, polytransesterification, and SSP have enabled PEF with a high molecular weight to be consistently and stably produced, it is still important to develop an alternative method of polymerization of PEF for further functionalization and minimization of side reactions. One interesting alternative is ROP from cyclic compounds consisting of FDCA and liner alkyl diols (Figure 9). ROP is advantageous as it can precisely control molecular weight by adjusting initiator and monomer ratio. Precise control of polydispersity can also be attained by reducing trans-esterification. In addition, various sequence structures can be prepared by copolymerization with other lactones, and end group functionalization. One study reported ROP of poly(butylene 2,5-furandicarboxylate) (PBF) [98]. In this report, cyclic oligomers of PBF are synthesized by reaction of furandicarbonyl dichloride (FDCC) and 1,4-butanediol in solution, and the obtained cyclic oligomers were used for ROP at 270 °C in a bulk state. The molecular weight of the obtained PBF was too low for practical applications, but the thermal properties were comparable to those achieved using conventional polymerization.

High molecular weight PBF and PEF were also obtained in another study [99]. The starting cyclic oligomers were prepared by reaction of FDCC and corresponding diols, and the remaining liner oligomer was carefully removed. The obtained cyclic oligomers were used for ROP, which was catalyzed by stannous octoate. The final molecular weight was 50,000 with *M*_w_/*M*_n_ of 1.4 for PEF and 65,000 with *M*_w_/*M*_n_ of 1.9 for PBF. Differences in the physical properties of PEF obtained using polycondensation/SSP and ROP have not been deeply studied yet, but these differences will lead to new applications of PEF.

### 4.2. High-Performance PLA from Modified Lactides

The functional groups in the main chains of polylactones such as methylene, ester, and ether control the properties and functionalities of these polylactones, but the functional groups in side chains also play an important role. A simple example of an effect from difference in side group structure is the difference in the thermal properties of polyglycolide (PGL) and PLA. The *T*_g_ and *T*_m_ of PGL are around 40 °C and 230 °C, respectively, and those of PLA are 55 °C and 170 °C, respectively. In addition, methyl substitution results in higher hydrophobicity, so the hydrolytic stability of PLA is higher than that of PGL. This indicates that a desired functionalization of PLA can be managed by optimum substitution of the methyl group of lactide for other functional groups to overcome the drawbacks of PLA, such as low *T*_g_ and transparency.

It has been proposed that *T*_g_ can be improved by substituting methyl for a bulky functional group, such as a phenyl group, and a phenyl-substituted lactide is practically reported by using naturally derived phenyl containing mandelic acid [100]. Mandelic acid is a biobased α-hydroxy acid that is widely used as a precursor for cosmetics, food additives, and other chemicals. Phenyl-substituted lactide can be synthesized by cyclic dimerization of mandelic acid, also called mandelide, and the reported *T*_g_ of polymandelide (PMA) is higher than 100 °C, which is high enough to be an alternative to high-*T*_g_ petroleum-derived amorphous polymers such as polystyrene. The reported ROP of mandelide is only applicable for *meso*-mandelide, which produces completely amorphous PMA, because the high *T*_m_ and poor solubility of *racemic* mandelide are not suitable for ordinal ROP in bulk or solution state.

Another interesting polymerization of PMA is ROP of phenyl containing 1,3-dioxolan-4-one (Ph-DXO) [101]. This method allows for control of the chiral structure of the final PMA by preparing homo-chiral Ph-DXO, as Ph-DXO with any chirality has moderate solubility.

In addition, ROP of cyclic *o*-carboxyanhydride can be used for synthesis of *isotactic* PMA. This report is the first about *isotactic* PMA for crystalline PMA, and its *T*_m_ is reportedly higher than 310 °C [102]. These thermal properties of PMA allow for new applications of biobased polymers.

Norbornene-substituted lactide also yields high-*T*_g_ PLA [103]. For norbornene substitution, l-lactide is brominated, and then an elimination reaction produces (6*S*)-3-methylene-6-methyl-1,4-dioxane, which is modified by DA reaction, producing norbornene modified lactide is produced. When the norbornene-modified lactide is involved in a ring-opening metathesis reaction, a polymer with narrow polydispersity and *T*_g_ of 192 °C is obtained.

**Figure 10:**
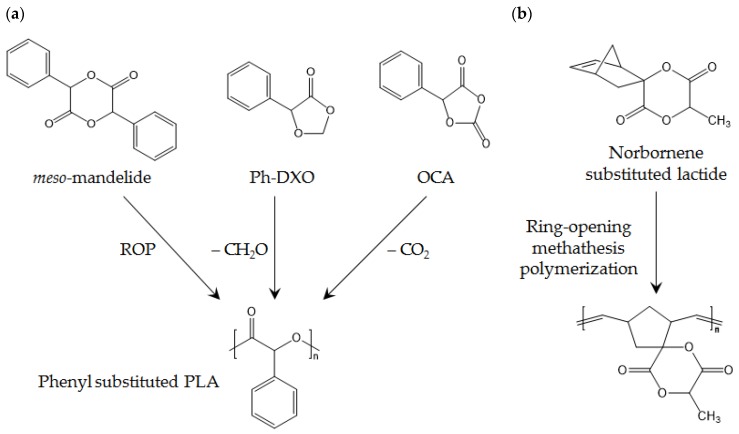
(**a**) Phenyl-substituted PLA; and (**b**) high-*T*_g_ polymer produced from norbornene-substituted lactide.

Substitution of methyl with a longer alkyl chain can be used to soften PLA by decreasing its *T*_g_. For example, ethylene-modified lactide results in *T*_g_ of 66 °C, *n*-hexyl-modified lactide results in *T*_g_ of −37 °C, and *iso*-butyl modified lactide results in *T*_g_ of 22 °C [104]. The ROP scheme of high-*T*_g_ PLA is shown in Figure 10, and the *T*_g_ values of the abovementioned substituted PLAs are listed in Table 8.

### 4.3. Terpen-Derived Biobased Polymers

Terpens are a class of naturally abundant organic compounds that are the main component of resins from a variety of plants, especially conifers. Terpens are used by plants for defense, deterring herbivores and attracting predators or parasites to those herbivores. In addition, some insects emit terpens from their osmeteria, such as termites and the caterpillars of swallowtail butterflies, also for defensive reasons. A variety of chemical modifications and functionalizations, such as oxidation, hydrogenation, and rearrangement of the carbon skeleton, can be applied to terpens, resulting in compounds called terpenoids. Both terpens and terpenoids are used in essential oils and fragrances for perfumes, cosmetics, and pharmaceuticals. The polymerizability of economically reasonable terpens and terpenoids is being studied, and there are interesting reports of biobased polyterpenes, especially in high-*T*_g_ polymers with excellent amorphous properties.

Pinenes are an important and widely known class of terpen. Polypinenes consisting of alicyclic hydrocarbon polymers comprised of β-pinene or α-phellandrene have high *T*_g_ (>130 °C), excellent transparency, and amorphous character [105,106,107]. In the early stage of development, polypinenes with high molecular weight are prepared by cationic polymerization using an optimum Lewis acid under polymerization conditions. However, the temperature required for polymerization (lower than −70 °C) is too low for industrial production.

**Figure 11:**
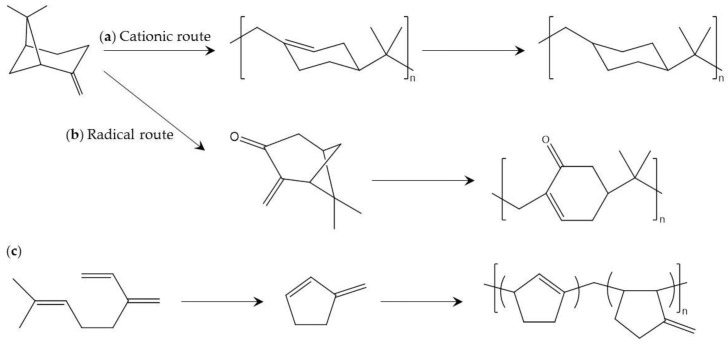
Production of polyterpenes from β-pinene using: (**a**) cationic polymerization [105]; and (**b**) radical polymerization [108]; and (**c**) production from myrcene [115].

Radical polymerization of modified pinenes has been reported to be an alternative to cationic polymerization of pinenes [108]. In this report, α-pinene is transformed into pinocarvone, which contains a reactive exo-methylene group involved in radical polymerization by chemical photo-oxidation and visible-light irradiation. Radical polymerization of pinocarvone is performed in relatively uncommon solvent to achieve high molecular weight and conversion as well as excellent thermal properties (*T*_g_ higher than 160 °C). The above mentioned cationic and radical polymerization methods are shown in Figure 11.

Limonene is classified as a cyclic terpen and the reason for the attractive smell of citrus fruits. Limonene is an optically active molecule, and its D-isomer is common in nature. D-limonene is widely used in the cosmetics and food industries. In addition to the economic value of limonene, it features high reactivity during radical polymerization in biobased polymer applications. High-*T*_g_ limonene homo-polymers can achieve excellent glass morphology [109]. The kinetics study in that report also indicates the possibility of high molecular weight and high polylimonene yield by optimizing the polymerization conditions.

Copolymerization of limonene and other vinyl groups containing monomers is an approach to synthesis of limonene copolymers [110]. One report presented a striking example of copolymerization of limonene and carbon dioxide to yield a high molecular weight polycarbonate [111]. In this report, limonene is converted to limonene-oxide, and polycarbonate obtained from copolymerization and thiol-ene coupling achieved excellent *T*_g_ (>150 °C). There are also interesting reports of chiral active polylimonens, as these show unique properties and stereocomplexability due to the interaction of chiral counterparts [112,113,114]. A interaction of l-configured and d-configured polylimonene carbonate forms a stereocomplex with *T*_g_ of >120 °C. Interestingly, the preferred crystallization of poly(limonene carbonate) occurs only in a stereocomplexed formation.

Myrcene is an organic olefinic hydrocarbon consisting of optically active carbon. β-myrcene is one of the main components of essential oils, but α-myrcene has not been found in nature and is used in extremely few situations. In industry, β-myrcene can be cheaply produced by pyrolysis of pinene. There are several interesting reports concerning polymers comprised of myrcenes and their derivatives. For example, myrcene can be converted to cyclic diene monomer, which produces an amorphous polymer (Figure 11) [115]. In addition, a copolymer of myrcene and dibutyl itaconate can be used for functionalized applications [116], and the low *T*_g_ of the copolymer is promising for biobased elastomer applications. The polymerization process and *T*_g_ of the featured polyterpenes are listed in Table 9.

### 4.4. Other Noteworthy Biobased Polymers

This section presents recent studies about new biobased polymers with physical properties that are superior to conventional petroleum-derived polymers. By utilizing naturally derived phenols, which contain aromatic rings, biobased liquid crystalline polymers (bio-LCP) can be developed. As the main chemical bonds of bio-LCP are ester bonds, hydroxy and carboxylic acid, biobased building blocks from natural phenols, can be used to produce bio-LCP. For example, 4-hydroxycinnamic acid (4HCA) is one important phenol that can be used to introduce liquid crystalline properties into polyesters via its aromatic function. 4HCA exists in plant cells that are intermediates of metabolites of the biosynthetic pathway of lignin. The mechanical properties of 4HCA-derived bio-LCP are superior to those of other commercialized biobased plastics, with a mechanical strength (σ) of 63 MPa, a Young’s modulus (*E*) of 16 GPa, and a maximum softening temperature of 169 °C [117,118].

One study reported a high-performance biobased polyamide with *T*_g_ of >250 °C [119]. This polyamide consists of repeating units from {(4,4′-diyl-α-truxillic acid dimethyl ester) 4,4′-diacetamido-α-truxillamide}. Monomers are prepared through conversion from naturally derived 4-aminophenylalanine, which involves UV coupling of cinnamic acid-derived vinyl groups from each monomer. Scientific investigation into monomer production and polymerization processes is still being performed, but this innovation indicates the possibility of development of super-engineering-grade polymers from naturally derived feed stock.

High-*T*_m_ biobased esterified poly(α-glucan) can undergo in vitro enzymatic synthesis [120]. Naturally available sucrose is used as the starting material for polymerization of linear poly(α-glucan), which is then enzymatically catalyzed and esterified on acetic or propionic anhydride. The *T*_g_ and *T*_m_ of acetated poly(α-glucan) are 177 °C and 339 °C, respectively, and the *T*_g_ and *T*_m_ of propionated poly(α-glucan) are 119 °C and 299 °C, respectively. The molecular weight of these polymers is higher than 150,000; therefore, it is expected that they have reliable mechanical strength when processed using the right procedures. The in vitro process is technically challenging in terms of scaling up and stabilizing production, but the promising thermal properties should be featured for future applications.

**Figure 12:**
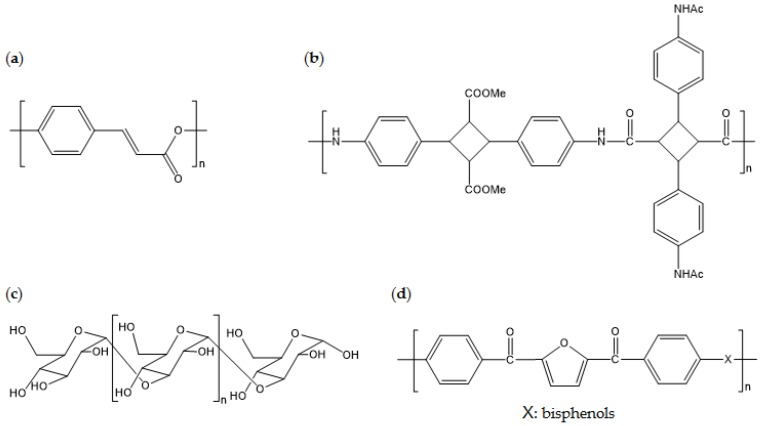
Chemical structures of: (**a**) poly(4-hydroxycinnamic acid); (**b**) poly((4,4′-diyl-α-truxillic acid dimethyl ester) 4,4′-diacetamido-α-truxillamide); (**c**) poly(α-glucan); and (**d**) poly(ether-ether ketone) consisting of FDCA derivatives.

Biobased poly(ether-ether ketone) (bio-PEEK) consisting of FDCA derivatives is a representative super-engineering biobased polymer. Bio-PEEK has a *T*_m_ of >300 °C, which is comparable to that of PEEK created from petroleum-derived resources [121]. Synthesis with TPA-derived biobased monomers is a way to replicate conventional PEEK. Thus, it is easily applicable to industrial processes as long as a supply chain of biobased furan derivatives are created. Figure 12 displays the chemical structures of the aforementioned biobased polymers.

## 5. Discussion

There have been constant and stable improvements in the production of biobased polymers (i.e., in the polymerization and refinery processes that yield biobased building blocks) in the past few decades. As a result, the application of biobased polymers has been expanded. In the early stage of development of biobased polymers, they were recognized as biodegradable polymers for temporary applications, which is still an important part of their applications. However, upgraded biodegradable polymers can now be used for general and engineering applications. These polymers as well as those that are analogous to conventional petroleum-derived polymers play an important role in further growth of biobased polymer applications. As the scaling-up of production of monomers for polymers that are analogous to conventional polymers has been successful, production of biobased polymers will also be scaled up. This will result in prices that are competitive with those of petroleum-derived polymers. Moreover, new biobased polymers comprised only of biobased building blocks, such as PEF and biobased polyamides, have unique and promising functionalities and applications. Thus, the goal of biobased polymer production is no longer to simply replace petroleum-derived polymers. Explorations into the topic will be accelerated by the development of high-spec engineering-grade biobased polymers, which have already been reported at the scientific level. We are confident that industrialization of these polymers will be discussed in the near future.

## Figures and Tables

**Table 1:** Development of biobased polymers and comparison with petroleum-derived polymers.

	Petroleum-derived polymers	Biobased polymers	
	Industry	Industrial approach	Scientific approach
Super-engineering applications	since 1960	not yet	since 2010
	PEEK, PSU, PES, PPS, PEI, PAI, LCP		bio-LCP, bio-PEEK (new generation)
Engineering/semi-engineering applications	since 1950	since 2010	since 2000
	Polyamide, POM, PC, PPO, PET, PTT, PBT, ultra-high MW PE, HIPS	bio-PET, bio-PTT, bio-PBT, bio-polyamide (analogous to petroleum-derived ones)	polyterpenes, PEF, bio-polyamide, sc-PLA (high *T*_m_), sb-PLA (high *T*_m_) (new generation)
General applications	since 1930	since 2000	since 1990
	PE, PP, PS, PMMA, PVC, ABS	PLLA (high-l content) reinforced PHAs, PHAs blends, succinate polymers, bio-PE/PP	sc-PLA (low *T*_m_), PHAs (super high MW), succinate polymers (upgrading from biodegradable polymers)
Biodegradable/biocompatible applications	since 1970	since 1990	since 1970
	PCL, PEG	PLLA (low-l content) PBS, PHAs, PGA, polysaccharides	PLA, PHAs, succinate polymers

**Table 2:** Poly(l-lactide) (PLLA) crystallization parameters [23].

l-Purity (%)	*M*_w_ (×10^5^)	Approximate value of growth rate of spherulite (μm/min) ^1^	*t*_s_ (min) ^2^	*t*_1/2_ (min) ^3^	*t*_e_ (min) ^4^	Crystallinity (%) ^5^
99.75	1.39	5.2	0.97	3.02	8.12	37.8
98.82	1.55	4.2	2.47	8.04	16.48	31.9
97.79	1.42	2.4	5.19	14.2	28.69	23.7

^1^ From analysis performed using polarized optical microscopy for isothermal crystallization at 130 °C (Approximate values from plot of Figure 2 in Reference [23]); ^2^ starting time of crystallization at 110 °C; ^3^ half-crystallization time at 110 °C; ^4^ ending time of crystallization at 110 °C; ^5^ Crystallinity after completion of crystallization at 110 °C.

**Table 3:** IR frequencies of amorphous, α’-form, and α-form PLLA [29].

	Amorphous (cm^−1^)	α’-Form (cm^−1^)	α-Form (cm^−1^)
ν_as_ (CH_3_)	2995	2997	2997
			3006
ν_s_ (CH_3_)	2945	2946	2946
			2964
ν (C=O)	1757	1761	1759
			1749
δ_as_ (CH_3_)	1454	1457	1457
			1444
δ_s_ (CH_3_)	1387	1386	1386
			1382

**Table 4:** Properties of commercial-grade Ingeo PLA [9].

Ingeo type	Application	MFR (g/10 min, 210 °C/2.16 kg)	*T*_m_ (°C)	*T*_g_ (°C)
2003D	extrusion, injection	6	145–160	55–60
3001D		22	155–170	55–60
3251D		80	155–170	55–60
3801X			155–170	45
4032D	film, sheet	7	155–170	55–60
4060D		10	-	55–60
6060D	fiber, non-woven	8	122–135	55–60
6252D		80	155–170	55–60
6752D		14	145–160	55–60

**Table 5:** Thermal and mechanical properties of representative PHAs [45].

	P3HB	P(3HB-*co*-20% 3HV)	P(3HB-*co*-12% 3HH)	Poly(4-hydroxybutyrate) (P4HB)	P(3HB-*co*-16% 4HB)
*T*_m_ (°C)	177	145	61	60	152
*T*_g_ (°C)	4	−1	−35	−50	−8
Tensile (MPa)	40	32	9	104	26
Elongation at break (%)	6	50	380	1000	444

**Table 6:** Chemical structures, suppliers, *T*_m_, and moduli of biobased polyamides [77,78,79].

Source	Chemical structure	Examples of commercial suppliers	*T*_m_ (°C)	Modulus (GPa)
Biobased	Polyamide 4	N.A.	265	
	Polyamide 4.6	DSM	295	
	Polyamide 4.10	DSM	250	1.3
	Polyamide 6.10	Evonik	206	2.1
	Polyamide 10.10	Arkema, Evonik	191	1.8
	Polyamide 11	Arkema	185	1.0
	Polyamide 12	Evonik	178	1.6
Petroleum derived	Polyamide 6	Chemical companies	218	3.0
	Polyamide 6.6	Chemical companies	258	2.5

**Table 7:** Comparison of the physical properties of PEF and PET [88].

	PEF	PET
Density (g/cm^3^)	1.43	1.36
O_2_ permeability	0.0107	0.114
CO_2_ permeability	0.026	0.46
*T*_g_ (°C)	88	76
*T*_m_ (°C)	210–230	250–270
E-modulus (GPa)	3.1–3.3	2.1–2.2
Yield stress (MPa)	90–100	50–60
Quiescent crystallization time (min)	20–30	2–3

**Table 8:** Chemical structure of modified lactides and their *T*_g_ values [100,101,102,103,104].

Modified lactide	*T*_g_ of Polymers (°C)
Glycolide	40
methyl glycolide(lactide)	66
ethylglycolide	15
hexyl glycolide	−37
isobutyl glycolide	22
cyclohexyl glycolide (meso)	96
cyclohexyl glycolide (iso)	104
meso-mandelide	100
Norbornene	192

**Table 9:** Polymerization process and thermal properties of polyterpenes [105,106,107,108,109,110,111,112,113,114,115].

	Polymerization	*T*_g_ (°C)
α-pinene	free radical	162
β-pinene	cationic	132
β-pinene	cationic	90
	cationic	130
limonene oxide	trans-carbonation	95
	trans-carbonation	114
limonene oxide/phthalic anhydride	ROP, ester	82
Myrcene/styrene	emulsion	−61
myrcene(3-methylenecyclopentene)	cationic polymerization	11
